# Parametric Modeling of Cochlear Electrode Arrays Using Design of Experiments and Finite Element Analysis (FEA)

**DOI:** 10.1155/abb/6620284

**Published:** 2026-06-22

**Authors:** Abdulaziz S. Alaboodi, Jamal Alsamri

**Affiliations:** ^1^ Department of Mechanical Engineering, College of Engineering, Qassim University, Buraidah, 51452, Saudi Arabia, qu.edu.sa; ^2^ Department of Biomedical Engineering, College of Engineering, Princess Nourah bint Abdulrahman University, P.O. Box 84428, Riyadh, 11671, Saudi Arabia, pnu.edu.sa

**Keywords:** biomechanics, cochlear implant, design of experiments, electrode arrays, finite element analysis, mechanical characterization, parametric benchmarking, platinum-iridium

## Abstract

Cochlear implant (CI) electrode arrays must navigate the delicate, spiraling microanatomy of the human cochlea. Optimizing their intrinsic mechanical properties is crucial for ensuring smooth surgical insertion and preventing extracochlear buckling. This study presents a parametric, bench‐type biomechanical evaluation of tapered cochlear electrode arrays, combining three‐dimensional (3D) finite element analysis (FEA) with a design of experiments (DOE) methodology. The array was modeled as a heterogeneous composite, comprising platinum‐iridium (Pt‐Ir) conductors embedded in a polydimethylsiloxane (PDMS) (silicone) matrix and evaluated as a free‐space cantilever under simulated surgical deflection conditions of up to 30°. This approach isolates the intrinsic bending stiffness and longitudinal column strength independent of complex tribological friction. A 15‐run factorial design varying apical radius, basal radius, and array length was utilized to quantify their interactive effects on tip deflection and reaction force. The FEA results demonstrated that across the parametric sweeps, maximum tip deflection ranged from 6.16 to 8.27 mm, while the reaction force varied between 1.096 and 4.66 mN. Peak Von Mises stress localized at the fixed basal end at 205.86 MPa, operating safely within the elastic limit of the composite’s alloy. Analysis of variance (ANOVA) revealed that array length is the dominant driver of tip deflection, whereas the basal radius governs reaction force due to its fourth‐power scaling of the area moment of inertia. Predictive regression models achieved adjusted *R*‐squared values approaching unity; as the experimental runs are derived from deterministic FEA simulations rather than stochastic physical trials, this near‐perfect fit reflects exact mathematical mapping of the response surface rather than real‐world physical variance. To validate this deterministic numerical framework, the outputs were successfully correlated against previously published experimental data using optical fibers as structural proxies under precision force measurement. Ultimately, these findings provide an efficient, predictive parametric design framework for benchmarking and comparing tapered electrode array geometries, utilizing flexural rigidity and reaction forces as fundamental proxies for safe surgical handling and structural trackability.

## 1. Introduction

Sensorineural hearing loss constitutes one of the most pervasive sensory deficits in the global population, with epidemiological forecasts projecting that one in four individuals will experience some degree of auditory impairment by the year 2050 [1]. Unsafe listening practices, aging populations, and genetic predispositions all contribute to this escalating public health concern. While conventional acoustic amplification via hearing aids is sufficient for mild to moderate deficits, severe‐to‐profound sensorineural hearing loss spanning a broad or complete frequency range frequently necessitates direct neural interfacing [2]. The cochlear implant (CI) has emerged as a profoundly transformative neural prosthetic device [3]. Unlike hearing aids, which amplify acoustic waves, CIs are designed to bypass damaged, malformed, or absent sensorineural structures, specifically the delicate inner hair cells of the organ of Corti, to directly stimulate the spiral ganglion neurons of the auditory nerve using electrical impulses [4, 5].

The functional architecture of a standard CI comprises external components (a sound processor and transmitting coil) and internal, surgically implanted components (a transcutaneous inductive receiver‐stimulator and an intracochlear electrode array) [6]. These components work together to provide a sense of sound to individuals with severe hearing loss, significantly improving their ability to communicate and interact with their environment [7]. The electrode array itself is an exquisitely engineered biomedical composite that must navigate the complex, spiraling microanatomy of the human cochlea. Inserted typically via the round window or through a surgically drilled cochleostomy, the array follows the spiraling trajectory of the scala tympani, a perilymph‐filled duct.

The fundamental bioengineering challenge of cochlear implantation resides primarily in the mechanical insertion process. The human cochlea is a highly fragile structure encased within the dense temporal bone. It contains exquisitely sensitive epithelial membranes, most notably the basilar membrane and Reissner’s membrane, as well as the highly vascularized stria vascularis, the delicate spiral ligament, and the osseous spiral lamina. Mechanical trauma during the surgical insertion of the electrode array can lead to immediate and irreversible loss of residual acoustic hearing, intracochlear bleeding, and the initiation of a severe, cascading foreign body response (FBR).

This localized inflammatory response to mechanical disruption and the introduction of foreign biomaterials often culminates in the formation of dense capsular fibrotic tissue and neo‐ossification (new bone formation) around the electrode array [8]. Fibrosis and ossification have deleterious consequences for the long‐term functionality of the implant. They physically tether the array, complicating any future explantation or reimplantation procedures, and, crucially, they act as high‐impedance electrical insulators. An increase in the electrical impedance between the electrode contacts and the target neurons necessitates higher stimulation currents to achieve the required charge density, which in turn causes rapid battery depletion, accelerates the electrochemical degradation of the electrodes, and induces electrical cross‐talk (current spread) between adjacent channels [9]. This cross‐talk severely degrades the spectral resolution of the perceived sound, making speech comprehension in noisy environments exceedingly difficult for the recipient. Consequently, optimizing the mechanical properties, geometrical design, and insertion dynamics of cochlear electrode arrays is of paramount importance to modern otologic surgery and neural engineering [10].

The mechanical behavior, biocompatibility, and long‐term reliability of a cochlear electrode array are strictly dictated by its composite material structure. Traditional and contemporary arrays are constructed as heterogenous composites, consisting of highly conductive, fatigue‐resistant metallic elements embedded within a highly flexible, biocompatible polymeric matrix [11].

To capture the complex nonlinear geometric mechanics, material heterogeneities, fluid dynamics, and dynamic boundary conditions inherent in cochlear implantation, researchers have increasingly relied upon sophisticated finite element analysis (FEA) frameworks [12, 13]. The evolution of FEA in this domain reflects a continuous drive toward higher anatomical fidelity and more accurate material modeling [7]. FEA models have also been developed to simulate traveling waves within the cochlea, contributing to improved electrode design and functionality [14].

Early computational efforts, notably the foundational work by Chen et al. [15], relied on simplified two‐dimensional (2D) FEA models to evaluate the trajectories and contact pressures of the nucleus straight electrode array. Even within the constraints of 2D, this early modeling provided critical insights, proving that arrays with a uniform stiffness profile generated the highest buckling stresses and peak contact pressures at the electrode tip. The 2D models conclusively demonstrated that a graded stiffness profile, achieved via tapering, was geometrically optimal for minimizing structural trauma, though the authors explicitly acknowledged the limitations of 2D approximations in capturing out‐of‐plane buckling and complex spiral geometry.

Modern FEA has wholly transitioned to high‐fidelity three‐dimensional (3D) modeling. Accurate simulation mandates precise geometrical reconstruction, typically derived from microcomputed tomography (*µ*‐CT) or ultrahigh‐resolution magnetic resonance imaging (*µ*‐MRI) scans of human temporal bones [16]. In a sophisticated 2023 study, Ren et al. [17] developed a comprehensive 3D FEA model by reconstructing cochlear structures from an average statistical shape model (SSM) of the human cochlea. This advanced spatial domain required fine discretization to resolve the extreme stress gradients occurring at the cellular level.

For the structural components of the electrode array and the surrounding bone, solid tetrahedral elements (such as Ansys Solid185) are extensively utilized, with a defined minimum element size of 0.05 mm. This specific discretization achieves strict mesh convergence and handles large deformation strains and hyperelastic material definitions without experiencing severe mesh distortion or volumetric locking. Furthermore, contemporary models increasingly incorporate fluid–structure interaction (FSI) to account for the dampening and resistive effects of the perilymph fluid inside the cochlea, utilizing specialized fluid elements (such as Ansys Fluid30). Ren’s 3D FSI models demonstrated that optimal array insertion parameters (lower Young’s modulus, effective tapering, and prebending) successfully minimized focal tip pressure while importantly preserving the natural tuning effect and basilar membrane displacement characteristics of the unimplanted cochlea, which is a key indicator for the preservation of residual acoustic hearing.

Building upon the preliminary finite FEA characterizations reported by Al Samri and Alaboodi [18], which established baseline deflection and reaction force responses for a limited set of tapered array configurations, the present study makes three distinct original contributions. First, this is the first study to apply a systematic three‐factor design of experiments (DOE) statistical framework to the parametric geometry of tapered cochlear electrode arrays, enabling simultaneous and statistically rigorous quantification of the individual and interactive effects of apical radius, basal radius, and array length on both tip deflection and reaction force across a clinically relevant design space. Second, the DOE‐FEA coupling generates novel closed‐form predictive regression models that allow engineers to estimate the mechanical performance of any array geometry within the parametric bounds instantaneously, without executing computationally expensive full 3D FEA simulations for each design candidate. Third, the study explicitly frames and justifies the bench‐type free‐space cantilever evaluation as a reproducible mechanical benchmarking methodology that isolates the intrinsic structural properties of the array—specifically its flexural rigidity and longitudinal column strength—independently of the complex tribological friction and wall‐contact dynamics encountered during actual cochlear insertion, thereby providing a controlled and reproducible basis for systematic design comparison.

## 2. Materials and Methods

### 2.1. Metallurgy of Platinum‐Iridium (Pt‐Ir) Alloys

The active metallic components of the array, comprising the exposed stimulation contact pads and the insulated longitudinal lead wires that connect the contacts to the receiver‐stimulator, are predominantly fabricated from a Pt‐Ir alloy, specifically the 90/10 formulation (90% platinum and 10% iridium by weight). Pure platinum exhibits exceptional biocompatibility, superior resistance to electrochemical corrosion during continuous pulsatile charge transfer, and highly favorable radiopacity, which is essential for postoperative radiographic confirmation of the array’s placement. However, pure platinum is mechanically soft and possesses a relatively low yield strength (typically exhibiting a Vickers hardness of merely 56 HV), making it highly susceptible to plastic deformation, bending, and eventual fatigue failure under the repeated microstresses of surgical handling and physiological tissue movement.

The addition of 10% iridium serves as a highly effective solid‐solution strengthening agent. Iridium significantly enhances the hardness, elastic modulus, and ultimate tensile strength of the matrix while preserving the requisite electrochemical inertness and biocompatibility required for long‐term implantation in neural tissue.

The processing history of the Pt‐Ir alloy dramatically influences its mechanical envelope, dictating its performance in vivo. Table 1 delineates the core physical, thermal, and mechanical properties of the Pt‐Ir 90/10 alloy across different processing states, reflecting the requirements for medical‐grade applications.

**Table 1:** Core physical, thermal, and mechanical properties of the Pt‐Ir 90/10 alloy across different processing states.

Material property	Pt‐Ir 90/10 (annealed)	Pt‐Ir 90/10 (cold‐worked)	Unit
Density	21.56	21.56–21.70	g/cm^3^
Melting point/solidus	1800–1831	1800–1831	^*o*^C
Young’s modulus (bulk)	200–201	200	GPa
Shear modulus	72.9–74	74	GPa
0.2% offset yield strength	445–490	896	MPa
Ultimate tensile strength	380–570	896–1000	MPa
Elongation to failure	8–25	2	%
Hardness (Brinell/HRA)	130–190 HB	55 HRA	—
Electrical resistivity	24.5–25.0	25.0	*μ* *Ω* · cm

As indicated by the empirical data, the transition from an annealed state to a cold‐worked (work‐hardened) state yields a massive increase in both yield strength and ultimate tensile strength, elevating the yield point from roughly 445 to 896 MPa. Medical‐grade wires utilized in cochlear arrays are frequently supplied in a highly finished, stress‐relieved, or cold‐worked condition to maximize their fatigue strength. This is critical because the lead wires often undergo significant mechanical manipulation during array fabrication, such as being shaped into complex wave patterns, and must withstand decades of continuous use without fracturing.

### 2.2. The Polymeric Matrix and Effective Bulk Modulus

The rigid metallic structures are encapsulated within a highly compliant, medical‐grade elastomeric carrier, universally composed of polydimethylsiloxane (PDMS), commonly referred to as silicone rubber. The silicone matrix serves a dual purpose: It acts as a flexible electrical insulator preventing short circuits between adjacent wires, and it provides a soft physical interface that protects the delicate cochlear endosteum from the hard, abrasive Pt‐Ir wires.

Because the electrode array is a composite structure, its effective, macroscopic mechanical properties, most notably its bulk flexural rigidity and Young’s modulus, are not simply equivalent to the 200 GPa modulus of the Pt‐Ir wires nor the highly compliant modulus of the silicone (which typically resides in the single‐digit MPa range). Instead, the effective stiffness is a highly complex geometric function determined by the volume fraction of the metal versus the polymer, the spatial distribution of the wires within the cross‐section, and the specific routing patterns employed.

For instance, modern array designs extensively utilize wave‐shaped (sinusoidal) wires rather than straight, linear wires. When an array containing straight wires is subjected to bending, the wires must endure direct axial tension and compression, thereby allowing the high 200 GPa modulus of the Pt‐Ir to dominate the bending stiffness [19]. Conversely, wave‐shaped wires act like microscopic springs; during array bending, the structural deflection is accommodated by the uncoiling or flattening of the wave amplitudes within the soft silicone matrix, drastically reducing the array’s effective flexural rigidity. This composite engineering allows the array to maintain high electrical conductivity and longitudinal column strength while exhibiting extreme transverse flexibility, a prerequisite for atraumatic insertion.

### 2.3. Geometrical Optimization and Commercial Configurations

The tonotopic organization of the human cochlea strictly maps high‐frequency acoustic signals to the basal region (near the round window) and progressively lower‐frequency sounds to the apical region (deep within the spiral). To achieve what is clinically termed complete cochlear coverage (CCC) and provide the user with the broadest possible spectral resolution of sound, an electrode array must ideally traverse deeper than one and a half turns, and in some anatomies, up to two full turns, into the scala tympani, without rupturing the basilar membrane or translocating into the adjacent scala vestibuli.

### 2.4. Tapered Versus Uniform Array Profiles

The macroscopic geometric profile of the array fundamentally dictates its insertion mechanics and its propensity to cause surgical trauma. Historically, earlier generations of electrode arrays utilized relatively uniform, cylindrical cross‐sections. However, longitudinal clinical outcomes and rigorous mechanical modeling have heavily favored tapered designs. In a tapered array, the basal end (proximal to the surgical opening and the middle ear) possesses a larger radius and inherently higher stiffness, while the apical end (the leading tip exploring the deep cochlea) features a highly reduced radius and significantly lower stiffness.

This functional gradient in geometric stiffness serves two distinct, critical biomechanical purposes. First, the softer, highly compliant tip minimizes the focal contact pressure exerted against the lateral wall and the delicate apical structures of the cochlear duct as the array navigates the tight radius of the apical turns. A uniform array maintains high stiffness at the tip, transferring massive buckling stresses directly into the tissue, greatly increasing the risk of mechanical penetration and subsequent permanent structural damage. Second, the thicker, stiffer basal section provides the necessary longitudinal column strength, often referred to as surgical “push ability,” to overcome the accumulating frictional resistance that develops as the array is advanced deeper into the cochlea [10, 20]. If the basal segment is engineered to be too compliant, the applied surgical force will cause the array to buckle extracochlearly or fold over upon itself within the basal turn, preventing full insertion.

### 2.5. Commercial Implementations and Anatomical Variations

Human cochlear duct lengths (CDLs) exhibit significant anatomical variation, typically ranging from 28 to over 36 mm [21]. Consequently, major CI manufacturers have developed extensive portfolios of electrode arrays with varying lengths, diameters, and shape philosophies to accommodate patient‐specific anatomies and diverse surgical preferences.

These philosophies generally bifurcate into two categories: lateral wall arrays and perimodiolar arrays. Lateral wall arrays are designed to rest gently against the outer wall of the scala tympani; this approach is widely considered highly atraumatic and is preferred for hearing preservation surgeries. Perimodiolar arrays, conversely, are manufactured with a precurved shape designed to hug the modiolus (the central bony axis of the cochlea). The objective of perimodiolar placement is to minimize the spatial gap between the stimulating electrodes and the target spiral ganglion cells, thereby reducing stimulation thresholds and minimizing current spread [22].

Table 2 synthesizes the dimensions and design philosophies of prevalent commercial electrode arrays.

**Table 2:** Dimensions and design philosophies of prevalent commercial electrode arrays.

Manufacturer	Array variant	Active length (mm)	Basal dim. (mm)	Apical dim. (mm)	Design philosophy
MED‐EL	FLEX 34	34.0	0.8	0.5 × 0.4	Extremely deep insertion, CCC, lateral wall
MED‐EL	FLEX 28	23.1 (28.0 total)	0.8	0.5 × 0.4	Standard lateral wall, highly flexible tip
MED‐EL	FLEX 20	20.0	0.8	0.5 × 0.3	Short insertion, optimized for hearing preservation
Cochlear	Slim straight	20.0–25.0	~0.8	<0.5	Straight, thin lateral wall
Cochlear	Contour Advance	~15.0–20.0	~0.8	~0.5	Precurved perimodiolar, stylet‐guided
Adv. Bionics	HiFocus SlimJ	23.0	0.76 × 0.56	0.55 × 0.26	Slim lateral wall, asymmetrical stiffness
Adv. Bionics	HiFocus Mid‐Scala	~17.0–18.5	0.70 × 0.70	0.50 × 0.50	Precurved, intended for central scala placement

The dimensional specifications highlight a rigorous bioengineering balancing act. Arrays must be sufficiently narrow to fit within the decreasing volumetric cross‐section of the scala tympani, which tapers significantly from the base toward the apex, yet possess adequate internal volume to securely house the 12–24 discrete electrical channels required for high‐fidelity speech coding. Advanced Bionics’ SlimJ array, for example, is explicitly designed to occupy less than 20% of the cross‐sectional volume of the scala tympani throughout its intended insertion depth. This volumetric minimization is highly intentional; it serves to preserve intracochlear hemodynamics, reduce mechanical displacement of the basilar membrane, and mitigate the severe trauma often associated with bulkier lateral wall electrodes.

### 2.6. Analytical Mechanics: Electrode Arrays as Tapered Cantilever Beams

**Figure 1:**
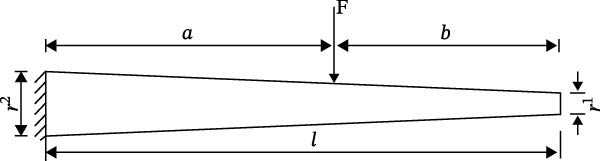
A cantilever beam with a fixed support, illustrating applied load *P* at distance a from the fixed end and parametric dimensions.

Prior to deploying computationally intensive numerical simulations, biomedical engineers frequently rely upon classical continuum mechanics to establish baseline behavioral metrics for electrode arrays. Due to its rigid anchoring at the surgical insertion tool (or the surgeon’s forceps) and its free‐floating projection into the cochlear fluid space prior to contact, the array can be effectively approximated mathematically as a cantilever beam subjected to transverse point loads (Figure 1). These localized loads represent the array making physical contact with the outer wall of the cochlea during its advancement.

For an idealized, purely elastic beam featuring a uniform, prismatic cross‐section and a fixed boundary at the basal end, the reaction force (*P*) generated by a transverse deflection at a specified interaction point is governed by standard Euler–Bernoulli beam theory. If a transverse load *P* is applied at a distance *a* from the fixed basal support, and the resulting maximum vertical deflection *δ*_max_ is evaluated, the generalized formulation is expressed as:

P=6EI⋅δmaxa23L−a.

Conversely, if one seeks to predict the maximum vertical displacement (*δ*_max_) resulting from a known applied point load *P* at distance *a* along a beam of total length *L*, the relationship is inverted:

δmax=P⋅a26EI3L−a.

Within these classical formulations, the parameter *E* represents the effective elastic modulus of the composite array, while *I* signifies the area moment of inertia. For a solid circular cross‐section based on a mean diameter (*d*), the moment of inertia is calculated as:

I=πd464.

While these analytical equations provide immediate, first‐order correlations establishing how an array’s diameter and flexural rigidity (EI) dictate its inherent stiffness, they exhibit severe limitations when applied to modern implant designs. Primarily, contemporary cochlear arrays are highly nonprismatic; they are intentionally tapered.

In a tapered beam geometry (Figure 1), the moment of inertia *I*(*x*) is no longer a static constant but rather a complex function of the longitudinal position *x*. Because the diameter *d* scales linearly in a standard conical taper, the moment of inertia *I*(*x*) scales with the fourth power of that decreasing diameter. This geometric reality causes an exponential, nonlinear reduction in flexural stiffness as one moves toward the apical tip. Consequently, utilizing classical prismatic Euler–Bernoulli beam formulas drastically overestimates the stiffness at the tip and entirely fails to accurately map the complex shear stress distributions and large‐deflection kinematics that evolve during multicontact cochlear insertion sequences. This severe mathematical limitation necessitates the transition to 3D FEA to accurately predict insertion trauma.

### 2.7. FEA of Tapered Array Mechanics and Stress Topologies

In a pivotal and highly detailed parametric study conducted by Al Samri and Alaboodi [18], a 3D FEA environment was constructed to map the exact deflection responses and reaction forces of varying tapered arrays operating under simulated surgical loads. Early work by this team indicated that while finite element results showed excellent agreement with experimental validations, there was initially perceived to be no significant difference in performance between purely tapered and uniform correctional electrodes in simplified scenarios. It should be noted that some early simplified cantilever evaluations exhibited discrepancies of 15% to 48% when compared to physical models. This variance is not primarily driven by friction but rather by the inherent mathematical limitations of homogenizing a complex metal‐elastomer composite array into a unified continuum rod with a single effective bulk modulus. However, expanding into rigorous 3D parametric arrays revealed the profound benefits of tapering.

In the expanded models, the array material was defined with a bulk, effective elastic modulus of 72.5 GPa and a Poisson’s ratio of 0.3, a figure carefully chosen to approximate the specific structural and volumetric blend of the rigid Pt‐Ir wires housed within the compliant silicone matrix. During the simulated load application, which incrementally enforced a deflection angle up to 30° (accurately mirroring the harsh mechanical curvature required to navigate the basal turn of the cochlea), the FEA elucidated the array’s internal stress topology.

**Figure 2:**
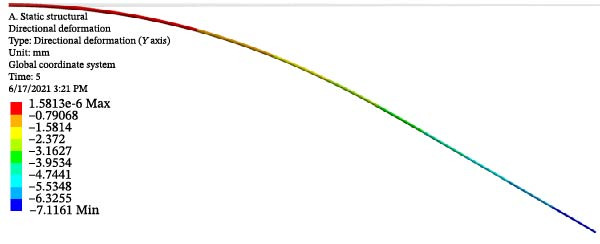
The FE deflection distribution of tapered arrays.

**Figure 3:**
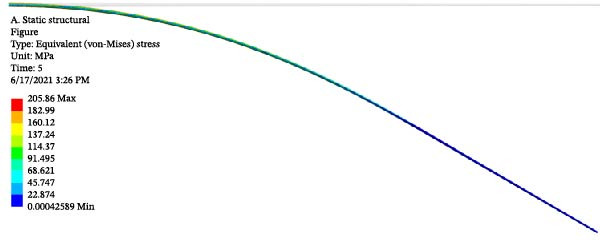
The FE Von Mises stress distribution of tapered arrays.

The models confirmed that the maximum spatial deflection systematically localizes at the free apical end, deflecting downward by up to 7.14 mm in the tested configurations (Figure 2). Crucially, the maximum Von Mises stress, the metric used to predict material yielding, consistently occurs at the fixed basal edge, where the array is anchored and the bending moment is maximized. In the specific parameter sweeps conducted by Al Samri and Alaboodi [18], the peak Von Mises stress reached 205.86 MPa (Figure 3).

**Figure 4:**
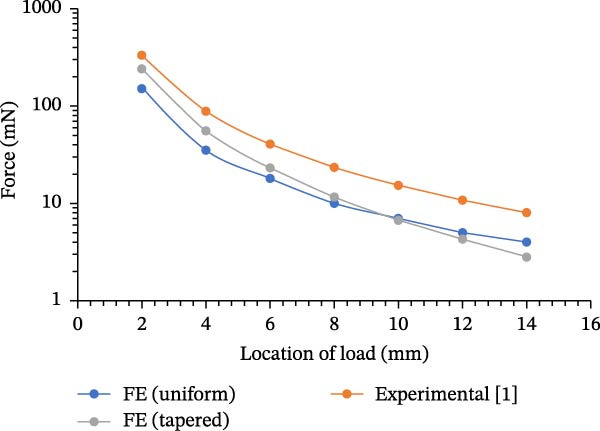
Reaction force versus load location of FE and experimental test (data reproduced from [18]).

This specific stress metric is profoundly significant when evaluated against the metallurgical data of the Pt‐Ir 90/10 alloy detailed earlier. Given that the absolute minimum yield strength of the alloy is 445 MPa (in its softest, fully annealed state), a peak Von Mises stress of ~206 MPa clearly indicates that the composite array operates well within its linear elastic domain during a standard 30° basal turn insertion. The material is subjected to less than half of its yield stress, representing a robust safety factor. This finding protects against catastrophic structural failure or localized plastic yielding (kinking) inside the cochlea. Plastic deformation during insertion is highly undesirable; a kinked array would permanently lose its designed shape, drastically increasing localized pressure on the cochlear wall, preventing smooth advancement, and making subsequent atraumatic extraction nearly impossible (Figure 4).

Furthermore, the FEA outputs definitively validate the necessity of the taper. Reaction force plots confirm a profound stiffness gradient in tapered designs: The array remains exceptionally stiff near the fixed end, preventing column buckling under axial load, and becomes highly compliant at the free end, allowing it to conform gently to the cochlear lateral wall rather than penetrating the endosteum lining.

### 2.8. Parametric Optimization Through DOE

While isolated FEA simulations are valuable, they represent single points of data. To systematically decode the intertwined influence of multiple geometric variables on the mechanical performance of the array, researchers leverage sophisticated DOE methodologies coupled with batch‐processed FEA. This combinatorial approach replaces isolated trial‐and‐error modeling with a statistically rigorous, highly efficient exploration of the entire geometric design space.

### 2.9. Factorial Matrix Construction

In the recent optimization studies led by Al Samri and Alaboodi [18], a specific three‐factor, multilevel factorial design was utilized, consisting of 15 unique experimental configurations executed within the FEA software. The independent input geometric parameters chosen for evaluation were the following:

1. Small radius (*r*_1_): The radius at the delicate apical tip varied strictly between 0.025 and 0.035 mm.
2. Large radius (*r*_2_): The radius at the rigid basal end varied between 0.05 and 0.07 mm.
3. Length (*l*): The total functional insertion length of the array varied between 18 and 22 mm.

**Figure 5:**
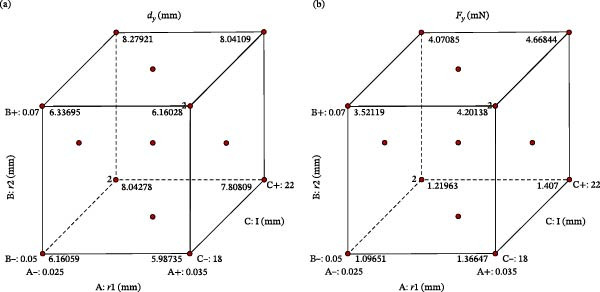
Graphical design of the factorial cube representation. (a) Factorial cube for the *d*_*y*_ response. (b) Factorial cube for the *F*_*y*_ response. High and low levels of each factor are denoted by (+) and (−), respectively.

The targeted output responses, acting as proxies for surgical handling and potential tissue trauma, were the maximum vertical deflection (*d*_*y*_, measured in mm) at the free end and the corresponding reaction force generated at the fixed edge (*F*_*y*_, measured in mN) as in Figure 4. Figure 5a,b visually represents the design using two squares (one for *d*_*y*_ and one for *F*_*y*_), each with four combinations at each corner. High and low levels for each factor are denoted by “+” and “−” signs, respectively. Table 3 extracts a representative subset of the factorial matrix, demonstrating how the parametric combinations drive the mechanical responses.

**Table 3:** Design of experiments (DOE) synthesized from the 15‐run factorial design.

Run	Small radius *r*_1_ (mm)	Large radius *r*_2_ (mm)	Length *l* (mm)	Deflection *d*_*y*_ (mm)	Reaction force *F*_*y*_ (mN)
1	0.030	0.06	20	7.116	2.406
2	0.025	0.06	20	7.226	2.206
4	0.030	0.05	20	7.000	1.279
5	0.030	0.07	20	7.210	4.137
8	0.025	0.05	18	6.160	1.096
14	0.025	0.07	22	8.276	4.080
15	0.035	0.07	22	8.041	4.668

*Note:* Data synthesized from the 15‐run factorial design demonstrating the parametric influence of array geometry on mechanical response.

## 3. Results

### 3.1. Statistical Deconvolution: Analysis of Variance (ANOVA)

To assess the statistical significance of these geometric factors and to quantify their individual and interactive effects on the array’s biomechanics, ANOVA was rigorously applied to the FEA outputs (Table 4).

**Table 4:** Analysis of variance (ANOVA) for deflection *d*_*y*_ (mm).

Source	Sum of squares	*df*	Mean square	*F*‐value	*p*‐Value	Significance
Model	9.07	9	1.01	81300.96	<0.0001	Significant
*r*_1_	0.1058	1	0.1058	8536.58	<0.0001	Significant
*r*_2_	0.1047	1	0.1047	8453.91	<0.0001	Significant
*l*	8.85	1	8.85	7.144E + 05	<0.0001	Significant
*r*_1_ · *r*_2_	5.864E‐06	1	5.864E‐06	0.4733	0.5221	Not significant
*r*_2_ · *l*	0.0018	1	0.0018	145.64	<0.0001	Significant

### 3.2. ANOVA for Deflection (**d**_**y**_)

For the deflection response (*d*_*y*_), the statistical model yielded an extraordinarily high *F*‐value of 81,300.96 with a corresponding *p*‐value of <0.0001, confirming a vast and undeniable statistical significance for the chosen geometric parameters.

### 3.3. Extracted ANOVA for Deflection

The ANOVA reveals that the array length (*l*) acts as the overwhelmingly dominant primary variable driving tip deflection, exhibiting a colossal *F*‐value of 714,400. This extreme sensitivity aligns perfectly with analytical beam theory, wherein deflection scales with the cube of length (*L* ^3^), making even millimeter adjustments to array length profoundly impactful on its flexibility and spatial trackability.

### 3.4. ANOVA for Reaction Force (*F*_*y*_)

A similar ANOVA analysis was conducted for the reaction force (*F*_*y*_) as it is shown in Table 5. Conversely, analyzing the reaction force (*F*_*y*_), which serves as a highly accurate proxy for the tactile resistance felt by the surgeon and the potential pressure trauma exerted upon the cochlear tissues, presents a different hierarchy of influence. The model for reaction force was highly significant (*F*‐value: 3745.70, *p*‐value <0.0001).

**Table 5:** Analysis of variance (ANOVA) for reaction force *F*_*y*_.

Source	Sum of squares	*df*	Mean square	*F*‐value	*p*‐Value	Significance
Model	21.36	9	2.37	3745.70	<0.0001	Significant
*r*_1_	0.4704	1	0.4704	742.30	<0.0001	Significant
*r*_2_	20.21	1	20.21	31886.97	<0.0001	Significant
*l*	0.2177	1	0.2177	343.53	<0.0001	Significant
*r*_2_ · *l*	0.0910	1	0.0910	143.55	<0.0001	Significant
$r_{2}^{2}$	0.2315	1	0.2315	365.38	<0.0001	Significant

In this paradigm, the large basal radius (*r*_2_) acts as the critical governor of reaction force, demonstrating an *F*‐value of 31,886.97, vastly outstripping the influence of length (*F*‐value: 343.53). As the basal diameter thickens, the area moment of inertia of the cross‐section expands geometrically to the fourth power. This drives an exponential surge in bulk flexural rigidity, resulting in a significantly higher reaction force transmitted back to the origin point during deflection.

### 3.5. Empirical Regression Modeling and Experimental Validation

The true power of this DOE framework lies in its ability to translate complex, computationally heavy, nonlinear FEA results into direct, predictive empirical formulas. For a biomedical designer seeking to rapidly assess a theoretical array’s performance without executing days of computationally expensive 3D contact FEA simulations, these coded response surfaces provide near‐instantaneous approximation. The generalized equations in terms of actual geometric factors were derived from the regression model:

dy=2.410.221.420.150.10.110.30+r1+r2+l+r1⋅r2+r2⋅l+r22,

Fy=7.110.100.100.940.200.20−r1+r2+l−r1⋅l+r2⋅l.

The precision of this predictive mathematical framework was rigorously validated experimentally. To strictly validate this predictive mathematical framework, the FEA outputs were correlated against previously published experimental data by Carland et al. [23]. In their benchmark study, physical optical fibers (acting as structural array proxies with varying diameters) were subjected to precision deflection and buckling force tests using a high‐resolution Mark‐10 force gauge under controlled, horizontal base‐secured conditions. The correlation between the FEA predictions, the regression equations, and the physical tests was exceptional. The regression model achieved an adjusted *R* ^2^ of 1.0000 for deflection and 0.9999 for reaction force while simultaneously exhibiting uniform, highly linear normal distributions on residual probability plots. It is essential to explicitly note that these near‐perfect *R*‐squared values are fully expected within this specific DOE framework. Because the data points are derived directly from deterministic numerical FEA simulations rather than stochastic physical trials, the statistical fit represents an exact mathematical mapping of the response surface rather than a measure of real‐world physical variance or experimental noise. This confirms that deterministic structural mechanics dominate array behavior and that these equations serve as robust design tools.

**Figure 6:**
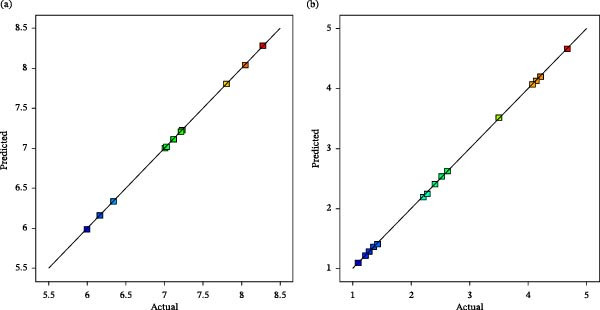
Predicted vs. actual values for (a) tip deflection *d*_*y*_ and (b) reaction force *F*_*y*_.

**Figure 7:**
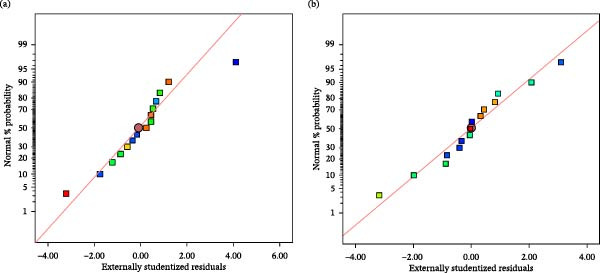
Normal probability plots of residuals for (a) tip deflection *d*_*y*_ and (b) reaction force *F*_*y*_.

**Figure 8:**
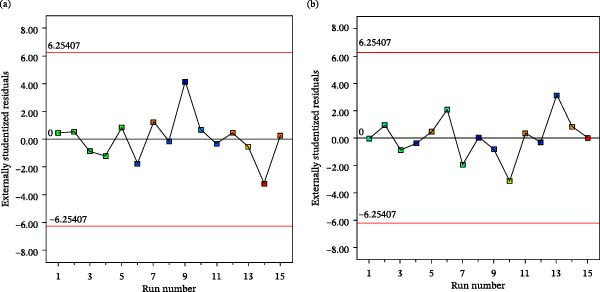
Residuals vs. run order for (a) tip deflection *d*_*y*_ and (b) reaction force *F*_*y*_.

**Figure 9:**
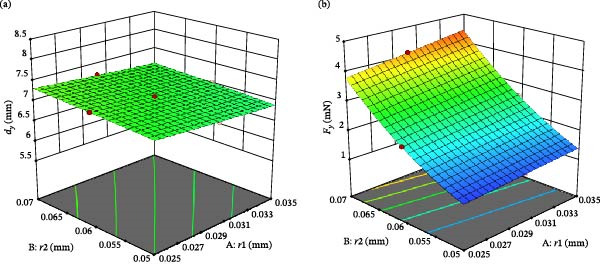
Three‐dimensional response surface of (a) tip deflection *d*_*y*_ and (b) reaction force *F*_*y*_ as functions of apical radius *r*_1_ and basal radius *r*_2_.

**Figure 10:**
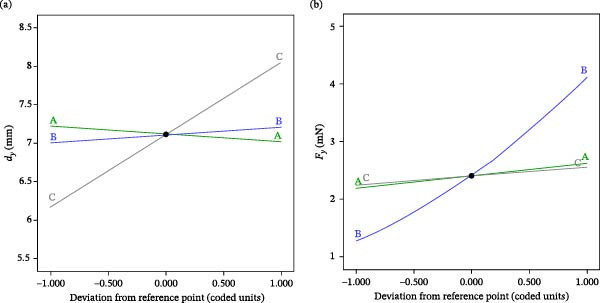
Factor interaction plots showing the effects of *r*_1_, *r*_2_, and *l* on (a) tip deflection *d*_*y*_ and (b) reaction force *F*_*y*_.

Figures 6–8 collectively validate the statistical quality of the regression models. Figure 6 confirms that predicted values align tightly with FEA‐simulated outputs for both responses, while Figure 7 verifies that residuals follow a normal distribution, a prerequisite for valid ANOVA inference. Figure 8 further confirms the absence of any run‐order trend or systematic bias in the residuals, ensuring the deterministic FEA simulations produced a smooth, well‐conditioned response surface. Figures 9 and 10 provide geometric insight into how the three design parameters interact to drive mechanical performance. The response surfaces in Figure 9 reveal the nonlinear, steep dependence of reaction force on basal radius *r*_2_, consistent with its fourth‐power scaling of the area moment of inertia, while tip deflection exhibits a more gradual, length‐dominated gradient. The interaction plots in Figure 10 confirm that the *r*_1_ × *r*_2_ interaction is negligible for both responses—consistent with the ANOVA results, whereas the *r*_2_ × *l* interaction exerts a significant combined influence on reaction force, underscoring the importance of co‐optimizing basal radius and array length in clinical electrode design.

## 4. Discussion

This study presents a parametric, bench‐type biomechanical evaluation of tapered cochlear electrode arrays, combining 3D FEA with a DOE statistical methodology to systematically quantify the effects of apical radius, basal radius, and array length on tip deflection and reaction force. The results confirm that the tapered geometric profile is mechanically superior to uniform cylindrical designs: The basal radius governs reaction force through its fourth‐power relationship with the area moment of inertia, while array length is the dominant driver of tip deflection, consistent with classical Euler–Bernoulli beam scaling. Peak Von Mises stress of 205.86 MPa remained well below the minimum yield strength of the Pt‐Ir 90/10 alloy (445 MPa in its annealed state), confirming that the composite array operates safely within its elastic domain under the simulated 30° surgical deflection loading.

The DOE‐FEA coupling generated closed‐form predictive regression models achieving adjusted *R* ^2^ values approaching unity. It is important to note that these near‐perfect statistical fits are fully expected within this deterministic framework: because all experimental runs are derived from numerical FEA simulations rather than stochastic physical trials, the *R* ^2^ values reflect exact mathematical mapping of the response surface and should not be interpreted as evidence of real‐world generalizability beyond the parametric bounds studied. Within those bounds, the regression equations provide engineers with a rapid, computationally inexpensive tool for estimating the mechanical performance of any candidate array geometry, validated against previously published experimental data by Carland et al. [23] using optical fibers as structural proxies.

Flexural rigidity and reaction force, as quantified here under bench‐type cantilever conditions, serve as fundamental proxies for two critical surgical handling properties: longitudinal column strength (resistance to extracochlear buckling during insertion) and tip compliance (the ability of the apical end to conform atraumatically to the cochlear lateral wall). The present study intentionally isolates these intrinsic structural properties from the tribological complexity of actual cochlear insertion, which involves distributed frictional wall contact, perilymph fluid displacement, and curvature constraints not modeled here.

Future work should extend this parametric DOE framework in three directions. First, incorporating frictional wall‐contact boundary conditions and cochlear geometry, informed by the tribological findings of [24], who demonstrated that sliding friction rather than hydrodynamic pressure is the dominant source of insertion trauma, would significantly increase the clinical relevance of the predicted force outputs. Second, the effect of internal structural modifications, such as the dummy‐wire configurations studied by Aşkın et al. [25], on the parametric stiffness landscape identified here represents a natural extension of the DOE factor space. Third, integrating real‐time strain sensing via embedded Fiber Bragg Grating optical fibers, as demonstrated by Carland et al. [23] and Gabardi et al. [26], into arrays designed using this benchmarking framework would enable objective intraoperative force monitoring, directly connecting the bench‐type mechanical predictions of this study to surgical outcomes. Together, these extensions will advance the transition from parametric mechanical benchmarking toward a fully validated, clinically predictive design methodology for cochlear electrode arrays.

## 5. Conclusion

This study establishes a parametric biomechanical framework for evaluating tapered cochlear electrode arrays through the integration of 3D FEA and a structured DOE methodology. The results demonstrate that basal radius predominantly governs reaction force through stiffness scaling, while array length is the principal driver of tip deflection, defining key mechanical trade‐offs relevant to surgical handling. The resulting closed‐form regression models provide a rapid and computationally efficient tool for predicting array performance within the defined parametric space, eliminating the need for repeated full‐scale simulations. By adopting a bench‐type free‐space cantilever configuration, this work isolates intrinsic structural properties, specifically flexural rigidity and longitudinal column strength, providing a reproducible benchmarking framework independent of complex cochlear tribology. While simplified boundary conditions were employed, the approach establishes a robust foundation for future integration of anatomical constraints, frictional interactions, and real‐time sensing. Collectively, these contributions support the rational, mechanics‐informed design of cochlear electrode arrays aimed at improving insertion control and reducing the risk of mechanical trauma.

## Funding

The authors extend their appreciation to Princess Nourah bint Abdulrahman University Researchers Supporting Project number (PNURSP2026R729), Princess Nourah bint Abdulrahman University, Riyadh, Saudi Arabia.

## Conflicts of Interest

The authors declare no conflicts of interest.

## Data Availability

Data are available upon reasonable request.
